# Gut microbiota and its metabolites promote painful diabetic peripheral neuropathy complicated with cognitive dysfunction in mice

**DOI:** 10.3389/fcimb.2025.1599954

**Published:** 2025-09-02

**Authors:** Junpeng Yang, Xin Lv, Ya Xu, Fenglian Huang, Xueli Yang, Xiaoyang Shi, Lingyun Zhao, Chenghong Liang, Danyu Wang, Yuanyuan Fang, Shasha Tang, Yalei Liu, Limin Wang, Xinru Deng, Xiaobing Wang, Huijuan Yuan

**Affiliations:** ^1^ Department of Endocrinology, Henan Provincial Key Medicine Laboratory of Intestinal Microecology and Diabetes, Henan Provincial People’s Hospital, Zhengzhou, Henan, China; ^2^ Department of Endocrinology, Henan Provincial Key Medicine Laboratory of Intestinal Microecology and Diabetes, People’s Hospital of Zhengzhou University, Zhengzhou, Henan, China; ^3^ Department of Endocrinology, Henan Provincial Key Medicine Laboratory of Intestinal Microecology and Diabetes, People’s Hospital of Henan University, Zhengzhou, Henan, China

**Keywords:** painful diabetic peripheral neuropathy, cognitive dysfunction, gut microbiota, metabolites, mice

## Abstract

**Introduction:**

Painful diabetic peripheral neuropathy (PDPN) is closely linked to cognitive dysfunction. The gut microbiota plays a pivotal role in the pathophysiology of diabetic neuropathy, but its contribution, along with related metabolites, to PDPN complicated by cognitive impairment remains poorly understood. This study aimed to explore the characteristics of gut microbiota and metabolites in *db*/*db* mice with PDPN and concomitant cognitive impairment, and to investigate the underlying mechanisms.

**Methods:**

Male homozygous *db*/*db* mice and their littermate *db*/*m* mice used as the research subjects. Thermal hyperalgesia and mechanical allodynia tests were applied to assess pain phenotypes, while the Morris water maze test was used to evaluate cognitive function. Immunohistochemistry was employed to measure intraepidermal nerve fiber density and nerve fiber markers, and Western blot analysis was used to detect pro-inflammatory cytokine levels. 16S rRNA gene sequencing of the V3-V4 regions was applied to analyze the gut microbiota structure, and LC-MS was used to analyze fecal metabolites.

**Results:**

At 12 weeks of age, *db*/*db* mice exhibited PDPN and cognitive deficits. The gut microbiota composition differed between the two groups, with LEfSe analysis identifying 38 key amplicon sequence variants (ASVs) enriched in *db*/*db* mice and 39 ASVs more abundant in *db*/*m* mice. Meanwhile, 398 metabolites that were significantly different between the two groups. Bidirectional mediation models indicated that Dl-lactate positively mediated the relationship between specific microbiota (*Muribaculaceae* (ASV243) and *Ruminococcus* (ASV149)) and thermal latency. In contrast, polygalic acid negatively mediated the relationship between *Muribaculaceae* and escape latency, as well as between *Ruminococcus* and thermal latency. These microbiota and metabolite changes were associated with elevated proinflammatory cytokine levels in the dorsal root ganglion (DRG) and hippocampus, respectively.

**Discussion:**

This study highlights the intricate relationship between gut microbiota, metabolites, and both PDPN and cognitive dysfunction in *db*/*db* mice. It also provides insights into potential mechanisms underlying the pathophysiology of these comorbidities, suggesting that modulation of the gut microbiota and its metabolites may offer new therapeutic strategies.

## Introduction

Diabetic neuropathy affects both the peripheral and central nervous systems. Diabetic symmetric polyneuropathy (DSPN) typically begins distally and progressively spreads proximally in a glove-and-stocking pattern (Yang et al., 2025). Around 30% of individuals with DSPN experience neuropathic pain, commonly referred to as painful diabetic peripheral neuropathy (PDPN) (Gylfadottir et al., 2020). PDPN significantly impacts patients’ daily activities, mood, and overall health-related quality of life. Cognitive dysfunction is increasingly recognized as a key comorbidity of diabetes mellitus, affecting various cognitive domains, including processing speed, executive function, and memory (Biessels and Despa, 2018). Numerous studies have shown that pain is closely linked to the cognitive system, with cognitive dysfunction also considered a comorbidity of chronic pain (Sloan et al., 2021). Pain perception involves a widely distributed network of brain regions and subcortical structures, collectively known as the “pain matrix”, and it can impair cognitive functions such as attention, memory, and learning (Todd et al., 2022).

Our previous study demonstrated that gut microbiota plays a causative role in PDPN, with gut barrier dysfunction, increased antigen load, and more severe systemic inflammation potentially contributing to the underlying mechanisms (Yang J. et al., 2023). Recent studies have emphasized the importance of microbiota homeostasis for maintaining gut health and modulating cognitive functions by regulating blood-brain barrier permeability, brain energy homeostasis, and synaptic transmission (Liu J. et al., 2020; Liu Z. et al., 2020). The mechanisms involved include metabolomic changes, such as the production of short-chain fatty acids and bile acids by the gut microbiota, which affect hippocampal mitochondrial biogenesis, energy metabolism, synaptic ultrastructure, and microglial inflammation (Liu J. et al., 2020). However, it remains unclear whether distinct gut microbiota and their associated metabolite profiles exist when PDPN and cognitive dysfunction co-occur, as well as how these factors influence pain, learning, and memory.

In this study, we investigate the characteristics of the gut microbiota and metabolomics in *db*/*db* mice with PDPN complicated by cognitive dysfunction. In addition, we use a bi-directional mediation analysis to explore the correlation network between key gut microbiota genera, metabolite levels, and clinical phenotypes, and discuss the underlying mechanisms.

## Materials and methods

### Reagents and chemicals

Eosin (Eosin Y solution HT110316) used by IENFD staining was purchased from Sigma-Aldrich (Darmstadt, Germany).The following antibodies were used in this study: The PGP9.5 antibody (ab109261) used in immunohistochemistry was purchased from Abcam (Cambridge, UK).The primary antibodies of MBP (Ab218011) and BDNF (Ab108319) used in immunohistochemistry were purchased from Abcam (Cambridge, UK).The primary antibodies of NF 200(18934 - 1-AP) was provided by Proteintech (Wuhan, China).The primary antibodies of NLRP3 (#15101), IL - 1β (#12426), caspase-1 (#24232), GAPDH (#97166) and tubulin (#2148) used in western blot were provided by Cell Signaling Technology(MA, USA).The secondary horseradish peroxidase-conjugated anti-rabbit/mouse antibodies(SAP - 9100) was purchased by Beijing Zhongshan Gold Bridge Biotechnology Co.(Beijing, China).

### Animals

All animal experimental procedures were approved by the Animal Experimental Center Committee of Zhengzhou University (Approval No. ZZU-LAC20220909) and were conducted in accordance with the committee’s guidelines. To avoid the influence of gender on the cognitive function, as well as the sex hormone status of female mice can cause fluctuations in metabolic and inflammatory levels, male *db/db* mice were used in this study (Cross et al., 2024; Wei et al., 2025). Male homozygous *db/db* mice (BKS.Cg-Dock7m+/+ Leprdb/J) and *db/m* (Dock7m+/+ Leprdb) heterozygotes, both from the same colony and aged seven weeks, were purchased from Gempharmatech (Certificate No. SCXK (Su) 2018 - 0008). The *db/db* mice and *db/m* mice were breed separately in a standard SPF (Specific Pathogen-Free) environment, under a 12-hour light/dark cycle, at a controlled temperature, with free access to food and water. After one week of acclimatization, the mice underwent assessments of blood glucose, body weight, and behavioral characteristics, which were monitored until they reached twelve weeks of age.

### Blood glucose and body weight

Blood glucose levels were measured weekly at the same time each afternoon between 8 and 12 weeks of age. A blood sample was drawn from the tail vein of each mouse and blood glucose was determined using a Yuwell glucometer (720, Jiangsu, China). Body weight was recorded every 7 days, at the same time of day, until the mice reached 12 weeks of age.

### Neurological function testing

The neurological pain function of mice was assessed by testing their thermal and mechanical sensitivity. The MWM test is an effective method for assessing spatial cognitive function. In this study, the Morris water maze was employed to evaluate spatial learning, spatial reference memory, and athletic ability in mice, thereby providing a comprehensive assessment of overall cognitive performance (Vorhees and Williams, 2006; Murayama et al., 2021).

### Thermal sensitivity

Thermal sensitivity was assessed following established methods using a thermal stimulation meter (YLS - 6B, Jinan, China) (Hargreaves et al., 1988). Prior to the procedure, adjust the hot plate temperature to approximately 55°C until it stabilizes. When a mouse is placed on a hot plate that is completely enclosed by transparent panels, the timer is activated. The timer is immediately halted once the mice exhibit behaviors such as rapid licking, hind paw retraction, or jumping. In order to avoid the feet of the rats being burned, if there was no response within 20 s, the latency was recorded as 20 s. Three measurements were taken for each animal at 15-minute intervals, and the average value for each mouse was calculated (Dixon, 1980; Jin et al., 2022).

### Mechanical sensitivity

Mechanical sensitivity was assessed using calibrated Von Frey filaments (Stoelting, Wood Dale, USA). The mice to be measured were placed in a box with a metal mesh and left for 15 – 30 min until the rats were acclimated to the unfamiliar environment. A series of von Frey filaments were selected (0.4, 0.6, 1.4, 2.0, 4.0, 6.0, 8.0, 15.0g, in total eight of filaments). Von Frey filaments were applied to the hairless part of the left palmoplanta of rats with a duration of action of 6 – 8 s. Lifting or licking of the foot on the stimulated side by the rats was considered positive. Each rat was tested three times, and the average was the final result. The 50% paw withdrawal threshold approach was adopted (Dixon, 1980).

### Morris water maze tests

Animals were tested in a circular tank with a diameter of 1.2 meters, located in a room equipped with extramaze cues (BW-MWM101, Shanghai, China). A platform, 10 cm in diameter, was submerged 0.5 cm beneath the water’s surface, with the water temperature maintained at an optimal level throughout the testing period. The experimenter divided the tank into four quadrants, labeled I, II, III, and IV, with the platform positioned in quadrant II. When water was added to the maze, its surface rose to a level approximately 0.5 – 1 cm above the platform. The environment remained quiet during the experiment to minimize external disturbances. The experiment lasted for five days, with the learning phase consisting of four trials per animal each day for a total of four days. Prior to the experiment, the mice were allowed to acclimate to the laboratory setting, and both the equipment and computer systems were calibrated to ensure the camera and tracking system were functioning optimally (Gu et al., 2024).

### Place navigation

Observe and record the routes taken by mice to locate and climb onto the platform, as well as the time required for this process, which is defined as the latency period. This method is employed to assess the acquisition of learning and memory abilities in mice through water maze performance.

### Spatial probe test

After the Place navigation experiment concluded, the platform was withdrawn and subsequently redeployed from the same entry point to record the number of times it traversed the original platform. It is used to assess the retention capacity of the spatial memory for the platform’s location in mice after they have learned to locate the platform.

### Immunohistochemistry analysis

#### Intraepidermal nerve fiber density measurement

A 2mm diameter skin biopsy instrument was used to horizontally drill the plantar skin of the mice. The sample that included both the epidermis and dermis was excised with ophthalmic scissors and was immediately fixed in 4% paraformaldehyde for the subsequent immunohistochemical analysis. The intraepidermal nerve fiber density (IENFD) was quantified by staining with PGP9.5 antibody. Eosin was used to counterstain the sections so as to make the fiber crossings at the dermoepidermal junction more prominent. The IENFD was calculated as the number of fibers per millimeter. This was done by counting the number of complete baseline nerve fiber crossings at the dermoepidermal junction (Chandrasekaran et al., 2019).

#### Dorsal root ganglion and sciatic nerve

The paraffin-embedded sections of the dorsal root ganglion underwent the process of deparaffinization with xylene and were dehydrated by being passed through a series of alcohol solutions with different concentrations. For antigen retrieval, either citrate buffer or EDTA buffer was employed. In order to inhibit the activity of endogenous peroxidase, the sections were treated with 3% H_2_O_2_ for a duration of 15 minutes. After that, they were incubated with diluted goat serum for 30 minutes so as to decrease nonspecific staining. Subsequently, the sections were incubated at 4°C overnight with the following primary antibodies: anti-NF 200 at a dilution of 1:300, anti-MBP at a dilution of 1:1000, and anti-BDNF at a dilution of 1:500.

#### Western blot

Firstly, 30 µg of protein from each sample in total was separated by utilizing SDS-PAGE (with a concentration range of 10 - 15%). Subsequently, the separated proteins were transferred onto a polyvinylidene difluoride (PVDF) membrane (0.22 µm pore size; Millipore, Billerica, MA, USA). The membranes were blocked for a period of 2 hours at room temperature in a solution of 5% non-fat milk in TBS-T. After being incubated overnight at 4 °C with primary antibodies, they were washed thoroughly with TBS-T. Next, they were incubated for 2 hours at room temperature with secondary horseradish peroxidase-conjugated anti-rabbit/mouse antibodies. Following another round of washing with TBS-T, the protein bands were visualized using enhanced chemiluminescence reagents (Millipore, Merck KGaA, Germany).

#### Statistical analysis

Statistical analyses were performed using GraphPad Prism 9.0. Data are presented as the mean ± standard error. The Shapiro-Wilk test was employed to assess the normality of the distributions. Differences across five time points were analyzed using a Two-Way Repeated Measures ANOVA. For comparisons between two groups, an unpaired Student’s t-test was applied when the data were normally distributed, and the nonparametric Mann-Whitney test was used when the data deviated from normality. Statistical significance was defined as ^*^ *P* < 0.05, ^**^ *P* < 0.01, ^***^ *P* < 0.001, and ^****^ *P* < 0.0001.

#### Gut microbiome analysis

Genomic DNA was isolated from mouse fecal samples through the utilization of the QIAamp PowerFecal Pro DNA Kit (QIAGEN, USA, 51804). Polymerase Chain Reaction (PCR) amplification targeting the V3-V4 region of the 16S rRNA gene was performed using the forward primer [5’-CCTACGGGNGGCWGCAG -3’] and the reverse primer [5’-GACTACHVGGGTATCTAATCC -3’] (Fang et al., 2021). The resulting amplicons were sequenced on the MiSeq platform, generating paired-end reads of 300 bp (Illumina, CA, USA).

The obtained reads were subjected to processing and analysis with QIIME2 version 2019.7, as detailed in reference (Xue et al., 2022). The “cutadapt” plugin integrated within QIIME2 was utilized to eliminate sequence adapters. To acquire the abundance and representative sequences of amplicon sequence variants (ASVs), the DADA2 algorithm was applied, as described in reference (Callahan et al., 2016). With the core - metrics - phylogenetic pipeline in QIIME2, a phylogenetic tree was built for the representative ASV sequences. Taxonomy assignment was carried out using the Silva database (release 132), as referenced in (Deng et al., 2022). All samples were randomly subsampled to an equal depth of 23,154 reads prior to further analysis.

The diversity plugins in QIIME2 were utilized to carry out α - diversity analysis and principal coordinate analysis (PCoA). For the permutational multivariate analysis of variance (PERMANOVA), with 999 tests being conducted, the R package vegan was employed. To identify the key ASVs that showed differences between the two groups, the Linear Discriminant Analysis Effect Size (LEfSe) method was used. Finally, the R package pheatmap was used to generate and visualize a heatmap representing the key ASVs.

#### Untargeted metabolomics analysis

The ultra-high performance liquid chromatography (UHPLC) system (1290 Infinity LC, Agilent Technologies) was coupled with a Quadrupole Time-of-Flight (QTOF) mass spectrometer (AB Sciex TripleTOF 6600) for the analyses. For hydrophilic interaction chromatography (HILIC) separation, samples were analyzed on a 2.1 mm × 100 mm ACQUITY UPLC BEH 1.7 μm column (Waters, Ireland). The mobile phase was composed of two solvents: A) 25 mM ammonium acetate and 25 mM ammonium hydroxide in water, and B) acetonitrile. The analyses were performed in both positive and negative ion modes using electrospray ionization (ESI).

For automatic tandem mass spectrometry (MS/MS) acquisition, the instrument was set to a mass-to-charge ratio (m/z) range of 25 – 1000 Da, with a cumulative scan time of 0.05 seconds per spectrum. Information-dependent acquisition (IDA) was employed for product ion scanning, and high-sensitivity mode was selected. The collision energy was fixed at 35 V ± 15 eV, with the deconvolution voltage set to ± 60 V.

Raw MS data (in. wiff scan files) were converted to MzXML format using ProteoWizard MSConvert, and feature detection, retention time correction, and alignment were performed using XCMS. Mass accuracy (< 25 ppm) and MS/MS data were employed for metabolite identification. The metabolites were annotated based on references from the KEGG database (https://www.kegg.jp/), BRITE, and the HMDB database (https://hmdb.ca/).

#### Bi-directional mediation analysis

To investigate the microbial features associated with both metabolites and neuropathy parameters, we first examined the relationship between neuropathic parameters and metabolites using Spearman correlation (FDR < 0.1). Next, we conducted a bi-directional mediation analysis, considering interactions (y = x + m + x × m, where y is the outcome, x is the variable, and m represents the mediator). This analysis, performed using the mediation function from the mediation package (version 4.5.0), aimed to infer the mediation effects of metabolites and the microbiome on phenotype parameters. The false discovery rate (FDR) was calculated using the Benjamini-Hochberg procedure.

## Results

### The phenotype of PDPN and cognitive dysfunction in *db/db* mice

**Figure 1:**
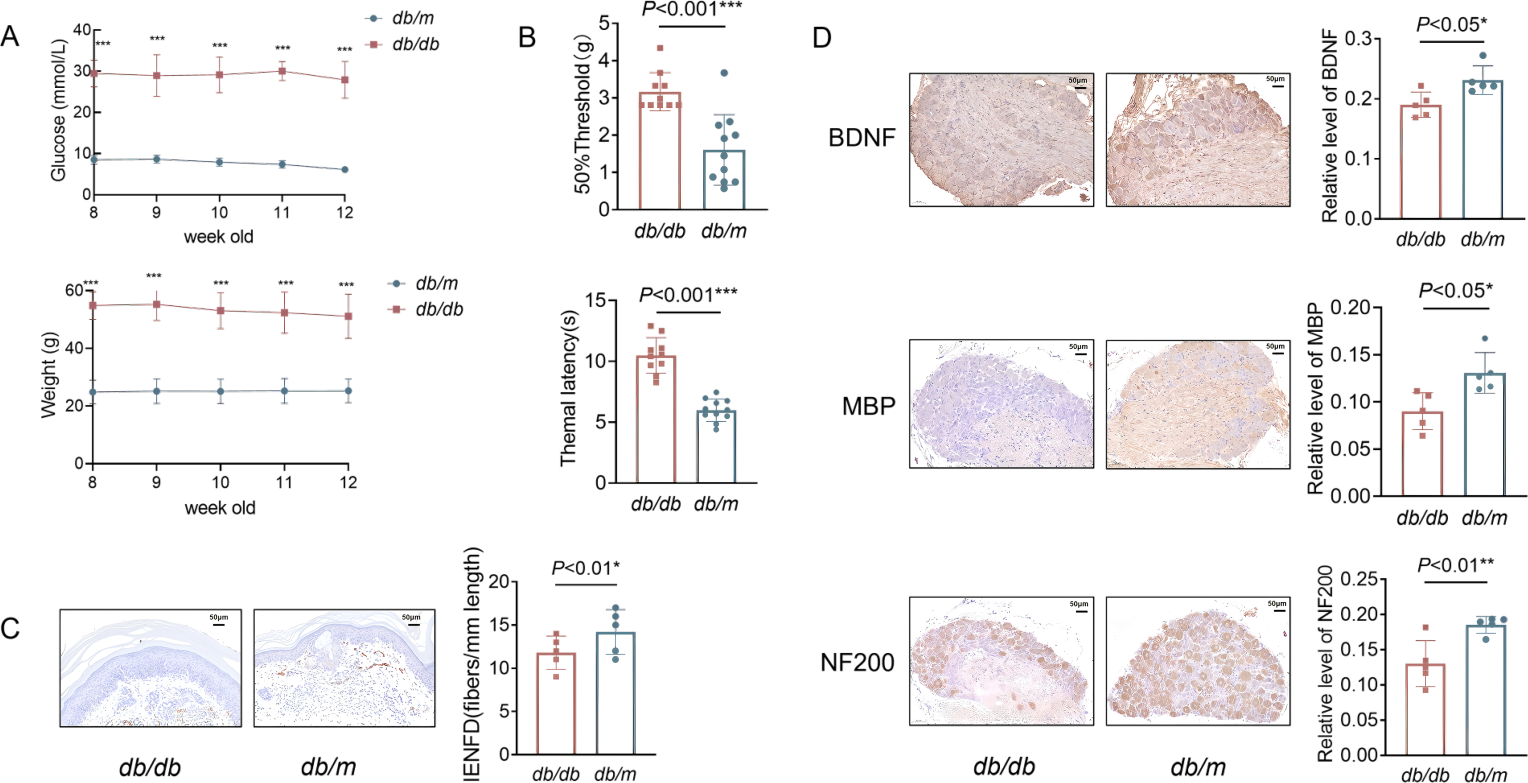
*db/db* mice exhibit higher levels of glycemia, weight and more severe peripheral neuropathy than *db/m* mice. **(A)** blood glucose levels and body weight from 8 weeks to 12 weeks. Two-way repeated-measures ANOVA followed by Tukey’s post was used for intra- and inter- group comparisons. **(B)** Mechanical sensitivity and thermal sensitivity. Immunohistochemical staining and integrated optical density (IOD) analysis of two groups mice for **(C)** intraepidermal nerve fiber density (IENFD, marked by PGP9.5) of the posterior plantar skin. Immunohistochemical staining of **(D)** dorsal root ganglion and IOD analysis for Brain derived neurotrophic factor (BDNF), myelin basic protein (MBP) and Neurofilament 200 (NF200). Date differences between two groups were compared with unpaired two-tailed Student t test **(B–D)**. *P < 0.05, **P < 0.01 and ***P < 0.001. For **(A, B)**, *db/db* group, n =10; *db/m* group, n = 11; For **(C, D)** n =5 per group. For **(C, D)** scale bars indicate 50 µm.

Although fasting glucose levels and body weight were significantly higher in the 10-week-old *db*/*db* mice compared to the *db*/*m* mice (**Figure 1A**), the phenotypic manifestation of PDPN did not appear until the mice reached 12 weeks of age. This was evident from a marked increase in the 50% threshold and thermal latency levels (**Figure 1B**), indicating that *db*/*db* mice exhibited significantly reduced mechanical and thermal sensitivity compared to the *db*/*m* group. Moreover, immunohistochemistry analysis revealed a significant reduction in intraepidermal nerve fiber density (IENFD) in the posterior plantar skin of *db/db* mice compared to *db/m* mice (**Figure 1C**), which is used to evaluate small fiber neuropathy and is associated with pain (Bonomo et al., 2020). Additionally, we observed significant decreases in the levels of NF200, MBP, and BDNF in the dorsal root ganglia (DRG) of the *db*/*db* mice compared to the *db*/*m* group (**Figure 1D**).

**Figure 2:**
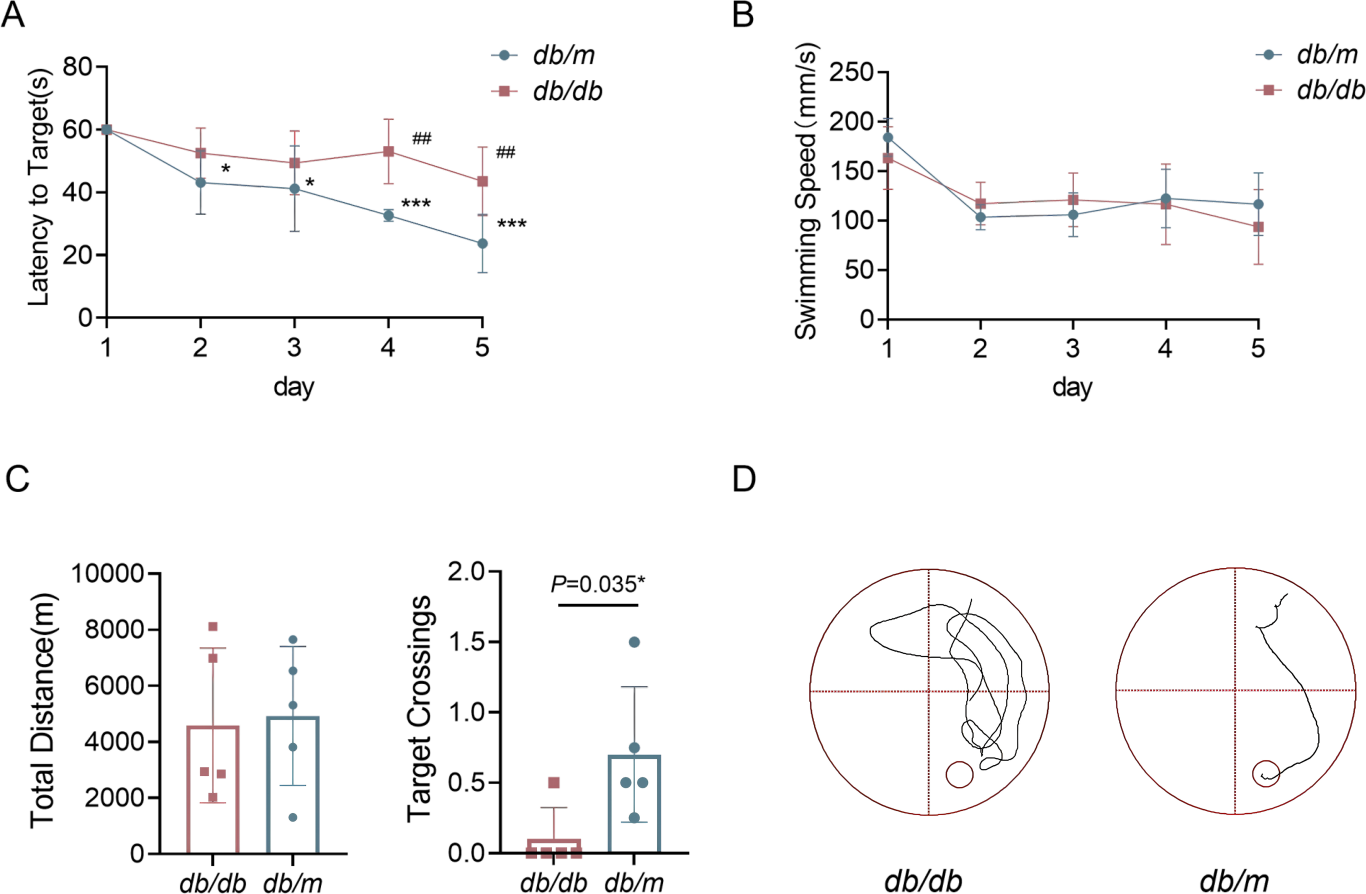
*db/db* mice displayed more severe deficits in spatial learning and memory in the Morris water maze (MWM). **(A–D)** Spatial learning and memory was assessed using the Morris Water Maze (MWM). **(A)** Five days learning trials assessed spatial learning via time to reach the target platform (shorter time = better performance). Two-way repeated-measures ANOVA followed by Tukey’s post was used for intra- and inter- group comparisons, ^##^ *P* < 0.01. One-Way RM ANOVA test was used to analyze differences between the five time point, *P < 0.05 and ***P < 0.001. **(B)**The average swimming speed of the two groups of mice in five days. Date differences between two groups were compared with unpaired two-tailed Student t test. **(C)** The total distance and the target crossings of two groups of mice in the last of days. Date differences between two groups were compared with unpaired two-tailed Student t test. *P < 0.05. **(D)** Spatial exploration experimental trajectory diagram of two groups mice. For (**A–C**), n=5 per group.

The Morris water maze (MWM) test was used to evaluate the spatial learning and memory abilities of mice. During the five-day training phase, *db*/*db* mice exhibited significantly longer escape latencies compared to the control group, particularly on days 4 and 5 (**Figure 2A**). The time for the mice to find the platform could reflect the learning and memory function of mice. A shorter time for the mice to find the platform means better learning and memory ability in mice. Swim velocity was evaluated in a MWM test to eliminate all bias due to visual and motor deficits. Throughout the MWM test, we did not observe any differences in movement speed between the two groups of mice (**Figure 2B**). In the subsequent spatial probe test, *db*/*db* mice demonstrated poorer cognitive performance than the controls, as evidenced by a lower number of target crossings (**Figure 2C**). However, no significant difference was observed in the total distance traveled between the two groups (**Figure 2C**). The corresponding swimming path maps are shown in **Figure 2D**. These findings indicate that *db*/*db* mice at 12 weeks of age display severe phenotypes of both PDPN and cognitive deficits.

### The characteristics of gut microbiota in *db/db* mice with PDPN and cognitive dysfunction

**Figure 3:**
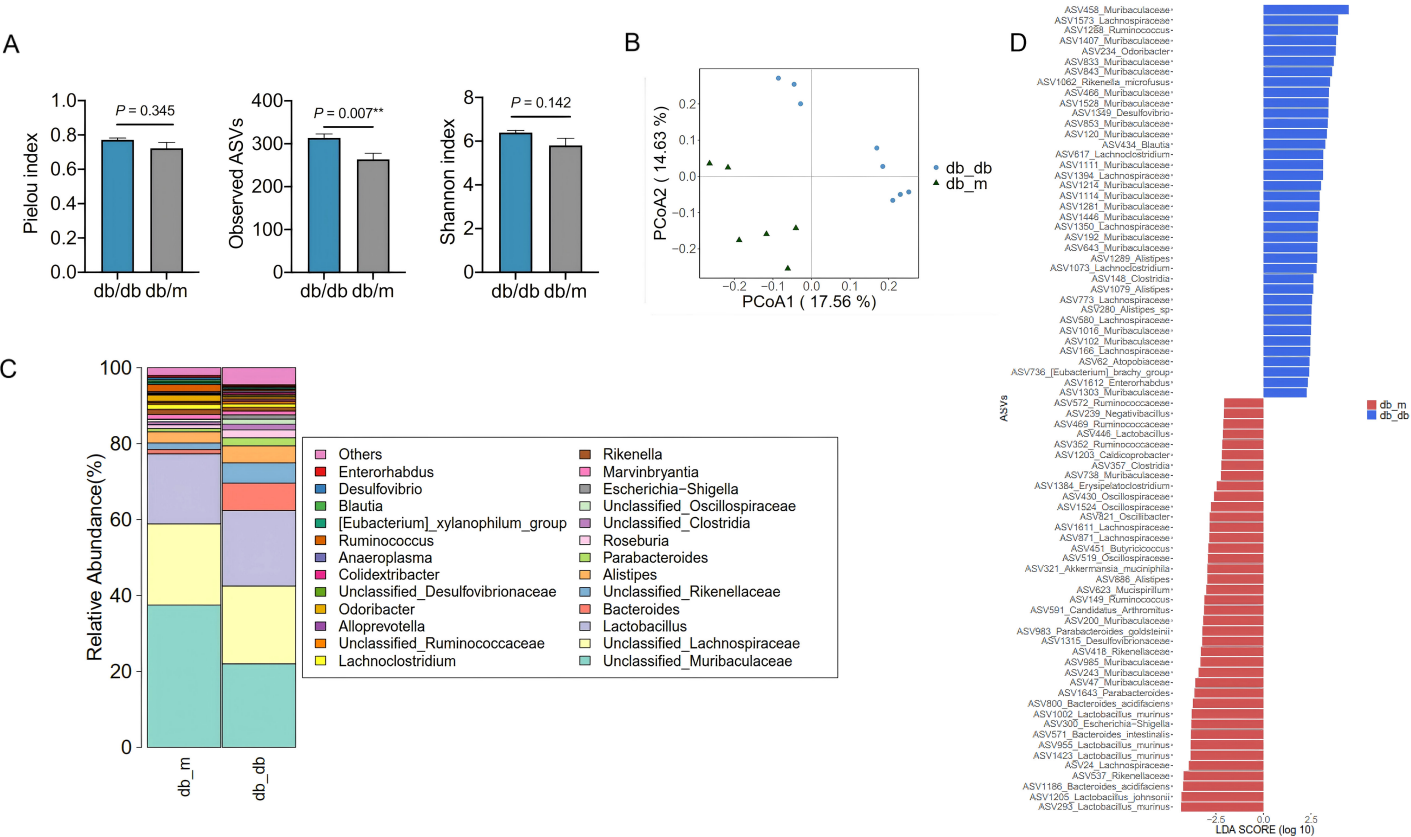
Identification of key gut microbiota in the context of PDPN with cognitive dysfunction in *db/db* mice. **(A)** The pielou index, observed ASVs and diversity (Shannon index) of the gut microbiota. **(B)** Principal coordinate analysis (PCoA) on the first two principal coordinates was performed based on the Jaccard distance. **(C)**The relative abundance of gut microbiota. **(D)** Identification of major amplicon sequence variants (ASVs) contributing to the differences in the gut microbiota using Linear discriminant analysis Effect Size (LEfSe). The comparison between *db/db* group and *db/m* group was tested with unpaired two-tailed Student t test. **P < 0.01. *db/db* group, n=8; *db/m* group, n=6.

We collected fecal samples from 12-week-old *db*/*db* mice and compared their gut microbiota with that of *db*/*m* mice, which maintain normal glucose levels. Analysis of the sequencing data from the V3-V4 regions of 16S rRNA genes revealed that the observed ASVs in the gut microbiota of *db*/*db* mice were significantly more abundant than those in the *db*/*m* group (**Figure 3A**). Principal Coordinate Analysis (PCoA) based on Jaccard distance showed a distinct difference in the overall gut microbiota structure between the *db*/*db* and *db*/*m* groups (**Figure 3B**). At the genus level, the gut microbiota abundance differed between the *db*/*db* and *db*/*m* groups (**Figure 3C**), and Linear Discriminant Analysis Effect Size (LEfSe) identified 78 key ASVs that exhibited significant differences between the two groups (**Figure 3D**). Notably, 40 ASVs were significantly more abundant in the *db/m* group compared to the *db*/*db* group. Among these, several ASVs, including three from *Lachnospiraceae* (ASV 1611, ASV 871, ASV 24), as well as one each from *Akkermansia muciniphila* (ASV 321) and *Alistipes* (ASV 886), are likely beneficial bacteria, as these ASVs in general are known to produce short-chain fatty acids (SCFAs). In contrast, 38 ASVs were more abundant in the *db*/*db* group, suggesting that these bacteria may be potentially harmful and pathogenic in conditions such as PDPN and CDI. Among these, 18 ASVs from *Muribaculaceae* (ASVs 1446, 1281, 1114, 1214, 1111, 1303, 643, 192, 120, 853, 1528, 466, 843, 833, 1407, 458, 102, 1016), and one each from *Ruminococcus* (ASV 1288) and *Desulfovibrio* (ASV 1349) were identified.

### The characteristics of metabolites and KEGG pathways in *db/db* mice with PDPN and cognitive dysfunction

**Figure 4:**
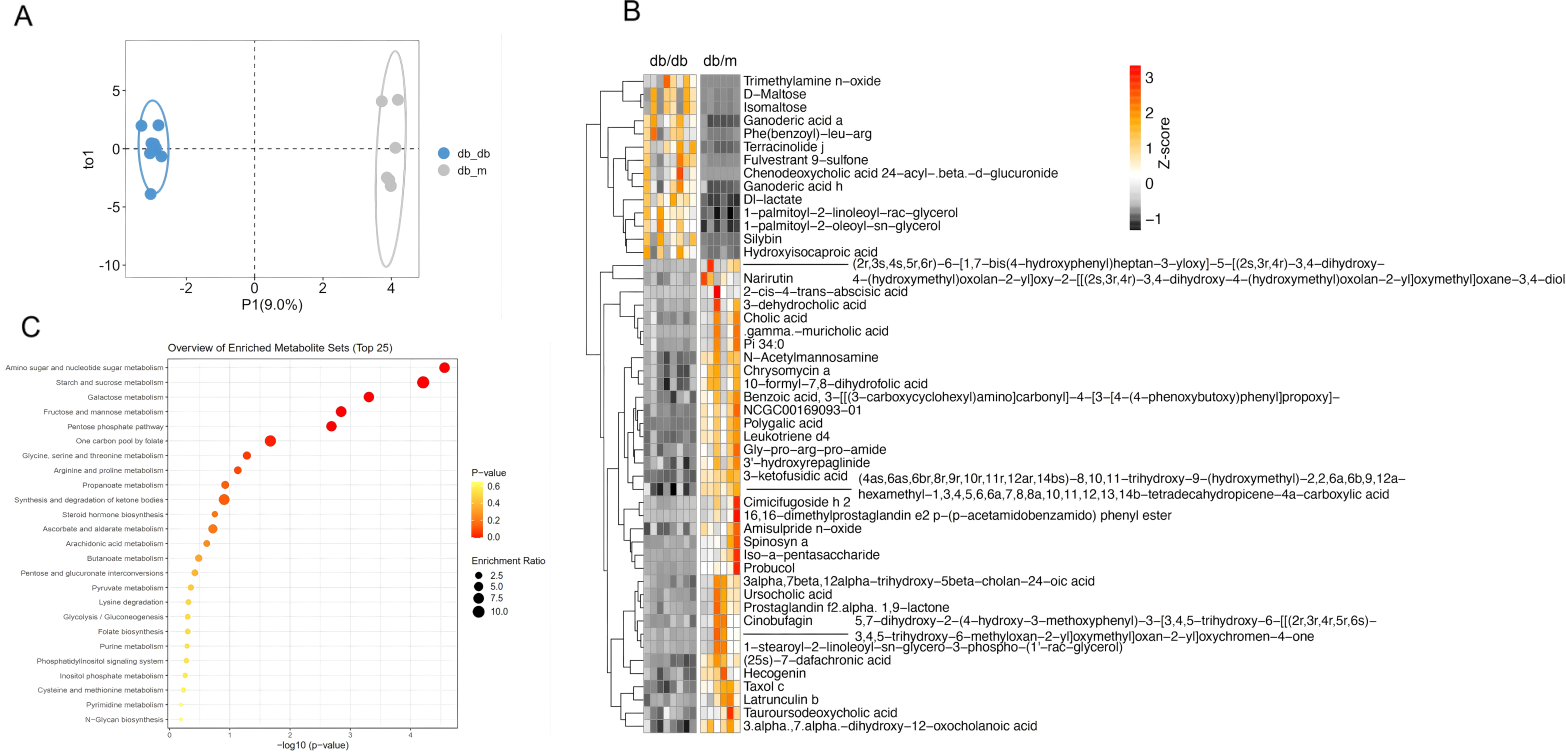
Identification of major fecal metabolites in the context of PDPN with cognitive dysfunction in *db/db* mice. **(A)** The OPLS-DA of fecal metabolites based on the Jaccard distance. **(B)** The heatmap of top 50 different fecal metabolites. **(C)** The KEGG pathway enrichment bubble of top 25 different fecal metabolites. *db/db* group, n=8; *db/m* group, n=6.

We then analyzed the fecal metabolites between the two groups and observed significant differences in the metabolite profiles of the mice with PDPN and cognitive dysfunction compared to those in the control group (**Figure 4A**). Using orthogonal partial least squares discriminant analysis (PLS-DA), we identified 398 metabolites that were significantly different between the two groups, with the top 50 metabolites shown in **Figure 4B**. Specifically, metabolites such as trimethylamine N-oxide, D-lactate, ganoderic acid A, beta-hydroxybutyrate, 3-dehydrocarnitine, acetophenazine, D-erythrose 4-phosphate, D-ribulose 5-phosphate, and 3,4-dihydroxyhydrocinnamic acid were significantly elevated in the *db*/*db* group compared to the *db*/*m* group. In contrast, metabolites such as (25S)-7-dafachronic acid, 20-hydroxyleukotriene B4, 6a,12a-didehydroamorphigenin, polygalic acid, and Gly-Pro-Arg-Pro-amide were found to be decreased. Additionally, we predicted the metabolic functions and presented the top 25 metabolic pathways showing expression differences between the two groups in **Figure 4C**. These pathways include those related to amino sugar and nucleotide sugar metabolism, starch and sucrose metabolism, galactose metabolism, the pentose phosphate pathway, and other metabolic processes.

### Correlation network between key genera in the gut, the levels of metabolites, and neuropathic phenotypes

**Figure 5:**
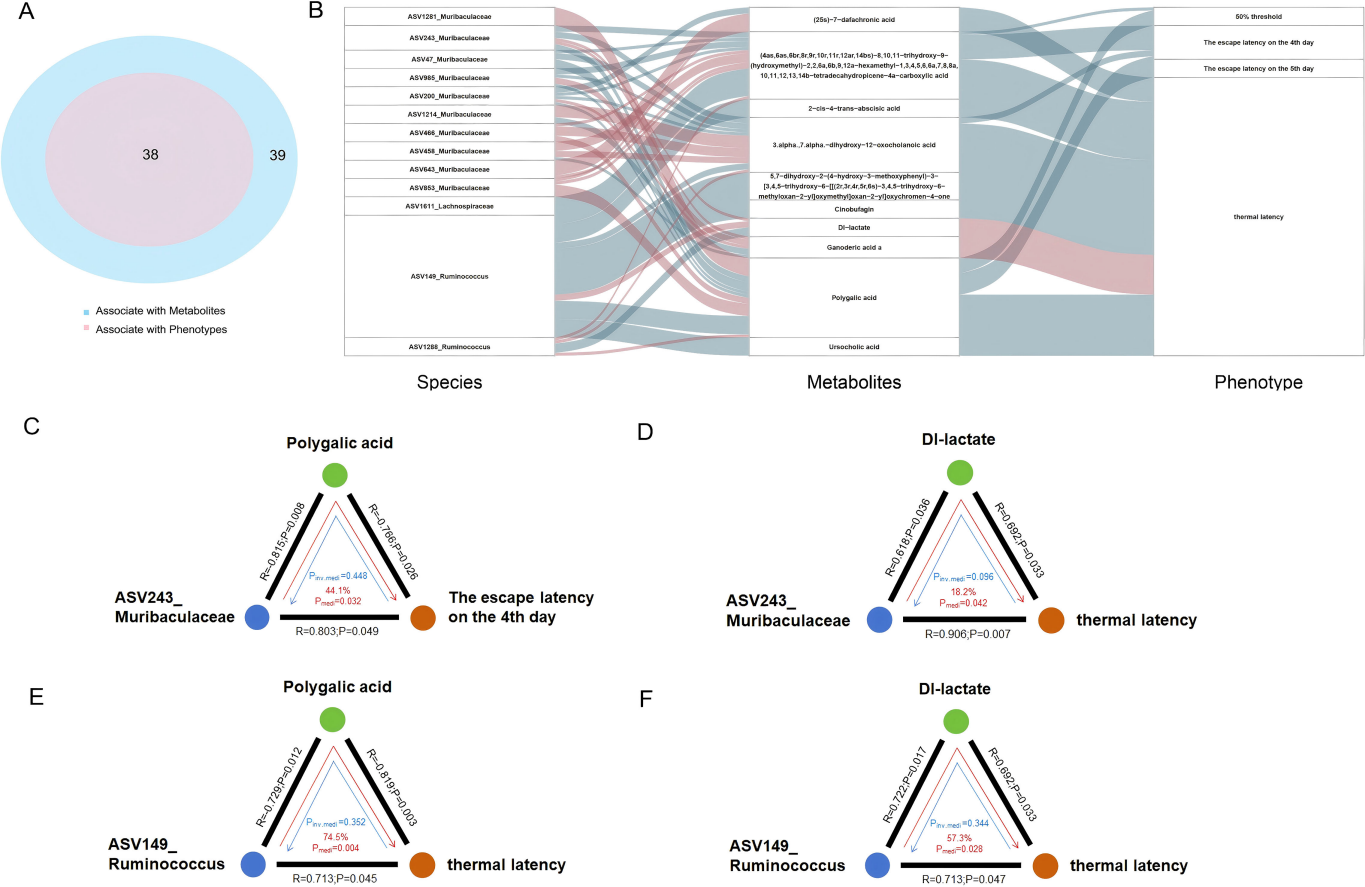
Mediation linkages among the gut microbiota, metabolites and phenotypes. **(A)** Venn plot of the number of microbial features that were associated with mice phenotypes and fecal metabolites, respectively. **(B)** Parallel coordinates chart showing the 51 significant mediation effects of fecal metabolites, the left panel shows the microbial factors, the middle panel shows the fecal metabolites, and the right panel shows phenotypes. The curved lines across panels indicate the mediation effects, while the red color represents positive correlation, blue color represents negative correlation. **(C)** *Muribaculaceae* (ASV243) causally contributed to the escape latency on the 4th day though Polygalic acid (P_mediatio_=0.032, 44.1%). **(D)** *Muribaculaceae* (ASV243) causally contributed to thermal latency through Dl-lactate (P_mediatio_=0.042, 18.2%). **(E)** *Ruminococcus* (ASV149) causally contributed to thermal latency through Polygalic acid (P_mediatio_=0.004, 74.5%). **(F)** *Ruminococcus* (ASV149) causally contributed to thermal latency through Dl-lactate (P_mediatio_=0.028, 57.3%). Inverse mediation was performed to check whether mice phenotypes can influence the microbiome through metabolites. The black lines indicate the Spearman correlations, with corresponding rho values and p values. The red arrowed lines indicate the microbial effects on phenotypes mediated by metabolites, with the corresponding mediation proportions and p values. The blue arrowed lines indicate reverse effects, with the corresponding inverse mediation p values.

To further investigate the role of fecal metabolites in the interaction between gut microbiota and host phenotypes, we constructed bidirectional mediation models (**Figure 5**). A total of 38 microbial features were found to be associated with neuropathic phenotypes, and 39 microbial features with metabolites (**Figure 5A**). To assess whether metabolites mediate the microbial impact on host phenotypes, we applied bidirectional mediation analysis, which revealed 51 significant mediation linkages (FDR < 0.05, and inverse mediation p-value > 0.05; **Figure 5B**; **Supplementary Table S1**). Most of these microbiota-mediator-phenotype linkages involved *Ruminococcus* and *Muribaculaceae*. Notably, the key metabolites of D-lactate and polygalic acid showed opposite correlations with thermal latency and escape latency on day 4. D-lactate, a product of glycolysis, was positively correlated with thermal latency, while polygalic acid was negatively correlated with escape latency. D-lactate overproduction has been linked to increased expression of pain-related ion channels, activation of satellite glia in dorsal root ganglion neurons, and an aggravated inflammatory response in a mouse model of STZ-induced diabetes (Rahman et al., 2016; Aghanoori et al., 2021). Polygalic acid, a major active constituent of Polygala tenuifolia, is believed to exert neuroprotective effects on cognitive impairment, partly by modulating cholinergic activity and reducing neuroinflammation (Zamudio-Cuevas et al., 2021). Our mediation analysis further suggested that *Muribaculaceae* (ASV243) may contribute to increased escape latency on day 4 and thermal latency by decreasing polygalic acid levels (44.1%, P_mediation = 0.032) and increasing D-lactate levels (18.2%, P_mediation = 0.042; **Figures 5C, D**). Additionally, we identified *Ruminococcus* (ASV149) as another harmful bacterium that may increase thermal latency by lowering polygalic acid levels (74.5%, P_mediation = 0.004) and elevating D-lactate levels (57.3%, P_mediation = 0.028; **Figures 5E, F**).

### The activity of NLRP3 inflammasomes in DRG neuro and hippocampus

**Figure 6:**
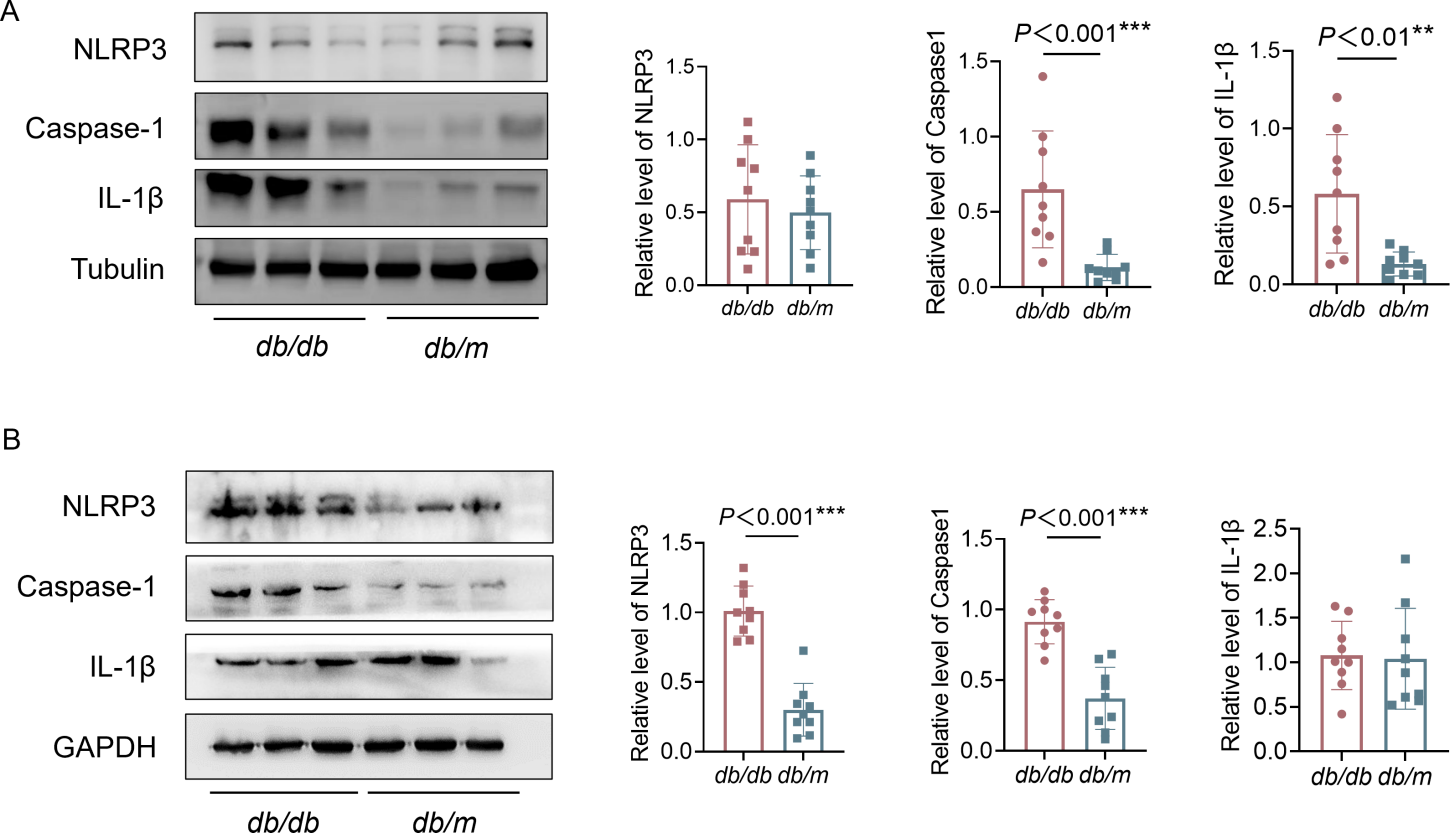
Protein levels of NLRP3, caspase-1, IL - 1β in DRG and hippocampus. **(A)** Protein levels of NLRP3, caspase-1, IL - 1β and Tubulin in DRG. **(B)** Protein levels of NLRP3, caspase-1, IL - 1β and GAPDH in hippocampus. Data are presented as mean ± SEM. n=3 per group. The comparison between the two groups was tested by Student’s t-test (two-tailed), **P < 0.01 and ***P < 0.001.

To further investigate the underlying mechanism linking gut microbiota and metabolites with both PDPN and cognitive dysfunction, we assessed the expression levels of NLRP3, caspase-1, and IL - 1β in the dorsal root ganglion (DRG) and hippocampus of mice using western blot analysis. The DRG serves as the convergence point for primary sensory neurons in the peripheral nervous system, while the hippocampus is a critical brain region involved in cognitive, emotional, and behavioral functions (Ma et al., 2022; Serger et al., 2022). At the protein level, we observed that IL - 1β and caspase-1 were significantly upregulated in the DRG of *db/db* mice compared to *db/m* controls. However, no significant differences in NLRP3 expression were found between the two groups in the DRG (**Figure 6A**). We then measured the levels of NLRP3, caspase-1, and IL - 1β in the hippocampus and found that NLRP3 and caspase-1 levels were significantly elevated in *db/db* mice relative to controls, while IL - 1β levels did not differ significantly between the two groups (**Figure 6B**).

## Discussion

Our research findings demonstrate that the gut microbiota and metabolites of *db/db* mice with PDPN combined with cognitive dysfunction were significantly changed, among which the Dl-lactate and Polygalic acid play a significant mediating role between the key microbiota *Muribaculaceae* (ASV243) and *Ruminococcus* (ASV149) with the phenotype of neuropathy. These microbial and metabolic changes were accompanied by elevated levels of proinflammatory cytokines in both the dorsal root ganglia (DRG) and hippocampus.

Previous studies conducted by our research group found that the prevalence of PDPN among hospitalized diabetic patients was as high as 51%, with more than one-third of these individuals experiencing moderate to severe pain (Yang et al., 2021). Furthermore, research has demonstrated that PDPN is closely associated with cognitive decline, including symptoms of anxiety and depression, as well as impaired sleep quality (Todd et al., 2022). Diabetes-related cognitive dysfunction carries a 50 - 100% increased risk of dementia. When these conditions co-occur, patients’ quality of life is significantly diminished (Shi et al., 2022). In this study, 12-week-old mice exhibited pain phenotypes manifested as elevated thresholds for mechanical allodynia and thermal hyperalgesia, reduced plantar nerve fiber density, and downregulated expression of neuronal markers NF200, MBP and BDNF in the DRG. Concomitantly, the mice showed compromised spatial learning and reference memory functions, as evidenced by prolonged escape latency and reduced target platform crossings in the MWM test. However, the underlying relationship between PDPN, cognitive decline, and the mechanisms involved remains unclear.

As the “second genome”, gut microbiota is closely associated with health status and disease progression throughout the human lifespan (Crost et al., 2023). It influences conditions such as diabetic cognitive dysfunction, inflammatory pain, neuropathic pain, and other pain-related diseases through the “gut-brain axis” and the “neuroimmuno-endocrine regulatory network”, with microbial metabolites like short-chain fatty acids (SCFAs) and cholic acid playing a key role (Zhao et al., 2018; Ma et al., 2022; Serger et al., 2022). In our previous study, gut microbiota from patients with diabetic sensorimotor polyneuropathy (DSPN) was transplanted into genetically diabetic mice, which had received antibiotic cocktail treatment in advance, exacerbating peripheral neuropathy. Moreover, our randomized controlled trial (RCT) involving fecal microbiota transplantation (FMT) demonstrated that transplanting gut microbiota from healthy donors was the sole trigger for improving nerve function and alleviating neuropathic symptoms in DSPN patients (Yang J. et al., 2023). Regarding cognitive function, intermittent fasting (IF) can enhance mitochondrial generation and energy metabolism gene expression in the hippocampus of the brain by altering the composition of gut microbiota, and then by altering the expression of metabolites such as SCFA and cholic acid, thereby exerting neuroprotective effects on db/db mice and improving behavioral disorders (Liu Z. et al., 2020).

In this study, we identified 18 ASVs of *Muribaculaceae* that were increased in db/db mice at 12 weeks of age. *Muribaculaceae* are highly abundant in the mouse intestinal tract, comprising 54.99 – 83.44% of the total *Bacteroides* content (Lagkouvardos et al., 2019). Our previous study found that the *Muribaculaceae* family in the gut and their translocation to the pancreas contributed to low-dose dextran sulfate sodium-induced diabetes (Yang X. et al., 2023). In this study, the analysis of bidirectional mediation models revealed that four ASVs of *Muribaculaceae* (ASV200, ASV243, ASV47, ASV985) were positively correlated with escape latency on the 4th day or thermal latency. Meanwhile, *Muribaculaceae* (ASV243) was associated with both, suggesting that *Muribaculaceae* may be a key bacterium involved in pain and cognitive decline in *db/db* mice. Meanwhile, we also found that *Ruminococcus* (ASV149) was significantly positively correlated with PDPN. *Ruminococcus* is a genus of bacteria in the class *Clostridia*, and some species of *Ruminococcus* are linked to Crohn’s disease and inflammatory bowel disease (Hall et al., 2017). Studies have shown that the abundance of *Ruminococcus gnavus* is closely associated with T2DM, and it is significantly positively correlated with insulin resistance, triglyceride, and cholesterol levels in T2DM patients. Furthermore, *Ruminococcus gnavus* is significantly increased in neuropsychiatric conditions such as cognitive dysfunction in the elderly, epilepsy, anxiety, and depression (Crost et al., 2023). Our study provides evidence that these two key bacteria may be detrimental and associated with PDPN accompanied by cognitive dysfunction in *db*/*db* mice.

Furthermore, our study also identified key metabolites mediating the effect of the microbiota and elucidated the underlying mechanism. In *db*/*db* mice with PDPN and cognitive dysfunction, the elevated metabolites are primarily involved in glucose metabolism pathways, such as Dl-lactate, D-erythrose 4-phosphate, and D-ribulose 5-phosphate. Growing evidence has revealed a novel mechanism of glucose metabolic reprogramming, including glycolysis and the pentose phosphate pathway, which plays a crucial role in diabetes and its chronic complications (Foltynie, 2019). In the STZ-induced diabetic mouse model, lactic acid is overproduced in dorsal root ganglia (DRG), leading to the activation of satellite glial cells and increased inflammation (Aghanoori et al., 2021). Our study demonstrated that Dl-lactate, an intermediate metabolite of glycolysis, mediates the relationship between two key bacteria, *Muribaculaceae* (ASV243) and *Ruminococcus* (ASV149), and thermal latency in *db*/*db* mice. However, it has certain limitations that exploring the causal relationships between microbiota/metabolites and neuropathic phenotypes solely through statistical analyses and support from previous literature. In future studies, we will conduct direct research on individual bacteria or metabolites to identify the specific microbiota-metabolite-neuropathy causal chain.

Numerous studies indicate that the gut microbiota and its metabolites participate in cognitive dysfunction and pian through the activation of the inflammasome (Zhao et al., 2023; Tang et al., 2025). In this study, we found the levels of NLRP3 and casepase-1 were significantly higher in the hippocampus, and the levels of IL - 1β and caspase-1 were significantly upregulated in the DRG of the *db/db* group compared to the *db/m* group in our study. Despite the absence of consistent changes in NLRP3, IL - 1β and caspase-1, likely due to factors such as the time points of detection, upstream/downstream regulatory mechanisms of the factors, and cell specificity, these findings nevertheless suggest that the inflammasome is activated in both DRG and hippocampal tissues (Wang et al., 2023). One study identified gut microbiota as a driver of chronic sleep deprivation and highlighted the NLRP3 inflammasome as a key regulator within the microbiota-gut-brain axis (Zhao et al., 2023). Besides that, bacterial metabolites such as bile acids can activate both signal 1 and 2 of the NLRP3 inflammasome in inflammatory macrophages (Hao et al., 2017). Study reveals that elevated lactate levels can mediate a pro-inflammatory response by activating the NLRP3 inflammasome (Fang et al., 2024). This finding underscores the role of gut microbiota in mediating cognitive dysfunction and pain via the Dl-lactate, and suggests its association with the activation of inflammatory responses in the hippocampus and DRG.

Moreover, we observed that several beneficial metabolites were significantly decreased in the *db*/*db* group. Notably, Polygalic acid, previously thought to have a protective effect on the nervous system, was found to be negatively correlated with cognitive function and pain in this study. While the current study focused exclusively on fecal metabolomic profiles, future research will integrate blood metabolomics with other omics approaches to enable a more systematic and comprehensive exploration of gut-neural axis interactions.

## Conclusion

In summary, this study demonstrates that PDPN co-occurring with cognitive dysfunction in *db/db* mice is associated with significant alterations in the gut microbiota and metabolites. We identified *Muribaculaceae* (ASV243) and *Ruminococcus* (ASV149) as key detrimental bacteria linked to neuropathic pain and cognitive decline. Crucially, Dl-lactate (an intermediate of glycolytic reprogramming) and Polygalic acid (a neuroprotective metabolite) were found to mediate the relationship between these microbiota and the neuropathic phenotype. These microbial-metabolic disturbances coincided with inflammasome activation in both DRG and hippocampal tissues.

This integrated gut-microbiota-metabolite-inflammasome axis, particularly involving Dl-lactate, underlies the comorbid pathogenesis of PDPN and cognitive dysfunction. Future research should focus on establishing direct causal chains and integrating multi-omics approaches for a deeper understanding of gut-neural interactions in diabetic complications.

## Data Availability

The datasets presented in this study can be found in online repositories. The names of the repository/repositories and accession number(s) can be found in the article/**Supplementary Material**.
